# Autism spectrum conditions in *hikikomori*: A pilot case–control study

**DOI:** 10.1111/pcn.13154

**Published:** 2020-10-13

**Authors:** Ryoko Katsuki, Masaru Tateno, Hiroaki Kubo, Keita Kurahara, Kohei Hayakawa, Nobuki Kuwano, Shigenobu Kanba, Takahiro A. Kato

**Affiliations:** ^1^ Department of Neuropsychiatry, Graduate School of Medical Sciences Kyushu University Fukuoka Japan; ^2^ Tokiwa Child Development Center Tokiwa Hospital Sapporo Japan; ^3^ Department of Neuropsychiatry Sapporo Medical University, School of Medicine Sapporo Japan

**Keywords:** autism spectrum conditions, *hikikomori*, modern‐type depression, narcissism, social withdrawal

## Abstract

**Aim:**

*Hikikomori*, a form of pathological social withdrawal, has been suggested to have comorbidity with autism spectrum disorder (ASD). This study aimed to clarify how characteristics of *hikikomori* are associated with ASD, including undiagnosed autism spectrum conditions (ASC), in clinical settings.

**Methods:**

A total of 416 clinical patients were recruited through the Mood Disorder/Hikikomori Clinic at Kyushu University Hospital. A total of 103 *hikikomori* cases and 221 clinical controls without *hikikomori* conditions were extracted using a semi‐structured interview, and completed a series of self‐rated scales, including the Japanese version of the Autism‐Spectrum Quotient (AQ‐J).

**Results:**

Compared to non‐*hikikomori* controls, *hikikomori* cases were more likely to have higher autistic tendency based on the AQ‐J. The cases showed more severe subjective depressive symptoms based on the self‐rated Beck Depression Inventory II, whereas no significant difference was found on interview‐based severity evaluation using the Hamilton Depression Rating Scale. Comparison within *hikikomori* cases based on the AQ‐J cut‐off score revealed that *hikikomori* cases with high ASC were significantly more likely to have higher traits of modern‐type depression, smaller social networks, and less social support.

**Conclusion:**

The present data suggest that *hikikomori* sufferers are more likely to have autistic tendency, and that *hikikomori* sufferers with high ASC may have much more difficulty in social communication and social interaction. In addition, those with high ASC may also have lower self‐esteem and higher complaint tendencies as aspects of modern‐type depression traits, which may relate to the occurrence of *hikikomori*. Thus, evaluating autistic tendencies is important for appropriate interventions in *hikikomori*. Further investigations should be conducted to validate our pilot findings using structured diagnostic systems of ASD.

*Hikikomori* is a form of pathological social withdrawal or social isolation that continues for more than 6 months with significant functional impairment or distress associated with the social isolation.^1^, ^2^ *Hikikomori* has been gathering attention, originally in Japan, since the late 1990s.^2^, ^3^, ^4^ Cases with *hikikomori* have since been reported in many other countries, such as South Korea, Spain, and the USA, making *hikikomori* an increasingly crucial global mental health issue.^5^, ^6^, ^7^, ^8^, ^9^, ^10^, ^11^, ^12^, ^13^

A variety of complicated biopsychosocial factors lie behind *hikikomori*,^14^, ^15^, ^16^, ^17^, ^18^ and *hikikomori* is known to be comorbid with various psychiatric disorders, including anxiety disorder, depression, personality disorder, Internet addiction, and autism spectrum disorder (ASD).^2^, ^15^, ^19^, ^20^, ^21^, ^22^

Regarding *hikikomori* and ASD, Tateno *et al*. surveyed a total of 1038 psychiatrists and pediatricians regarding their perceptions of *hikikomori*.^21^ Both groups responded that almost one‐fifth of their *hikikomori* patients could be diagnosed with F8 (Disorders of Psychological Development), such as pervasive developmental disorders (PDD) as the ICD‐10‐based^23^ psychiatric diagnosis. Kondo *et al*.^19^ reported that 31.8% of 183 *hikikomori* sufferers who attended mental health welfare centers had developmental disorders, such as mental retardation and PDD, as the DSM‐IV‐based^24^ psychiatric diagnosis. Kondo *et al*. have also reported the features of persons with high‐functioning PDD who were in *hikikomori* conditions: (i) they have strong anxiety and fear in novel situations and social situations; and (ii) in their childhood they showed inconspicuous PDD tendency while they had obvious symptoms, such as anxiety, fear, and food selectivity.^25^, ^26^ Although there are several studies on the relation between *hikikomori* and ASD, little empirical research has been conducted.

Modern‐type depression (MTD) is another novel psychopathological condition that started to gain attention at around the same time as *hikikomori*. Since around 2000, young adults with depressive symptoms whose premorbid personalities are different from the traditional type have increasingly been reported in Japan.^7^, ^27^, ^28^, ^29^, ^30^, ^31^ This novel depressive condition is currently called ‘Shin‐gata/Gendai‐gata utsu‐byo; modern‐type depression’ and is characterized by excessive complaints of depressive feelings in stressful settings, such as the workplace or school, and people around such individuals tend to regard them as having exaggerated reactions. In addition, an extrapunitive tendency is regarded to be one of the outstanding features of MTD.

We have proposed that the commonality between *hikikomori* and MTD is the social avoidance tendency, such as avoiding potential psychological attack from others, and that the prolongation of MTD may lead to occurrence of *hikikomori*.^32^, ^33^, ^34^ Recently, we developed the 22‐item Tarumi's Modern‐Type Depression Trait Scale: Avoidance of Social Roles, Complaint, and Low Self‐Esteem (TACS‐22), which evaluates premorbid personality traits of MTD.^35^ Based on a comparison in MDD patients with and without *hikikomori* conditions, we also found that MDD patients with *hikikomori* showed significantly higher scores on the TACS‐22 than the latter group.^36^

Interestingly, Sawada^37^ and Abe^38^ have suggested that MTD is related to ASD: Depressive patients who claim to have depression may include persons with PPD, and characteristics of MTD can be understood in the context of ASD traits. As an example, situation‐dependent depressive state may associate with excessive concentration and blaming others may relate to difficulty in social interaction and perseveration. However, no empirical research between MTD and ASD has been conducted.

Until now, as shown above, the deeper underlying relations between *hikikomori* and ASD (including undiagnosed autism spectrum conditions [ASC]), and their associations with MTD have not been examined thoroughly. Therefore, as a pilot study, we herein tried to clarify the relation between *hikikomori* and ASC in our clinical settings. Moreover, we explored characteristics such as premorbid personality traits relating to MTD, psychiatric symptoms, and social networks among *hikikomori* sufferers with higher and lower ASC.

## Methods

### Setting and procedures

All *hikikomori* cases and clinical controls were recruited through the Mood Disorder/Hikikomori Clinic in the Department of Neuropsychiatry at Kyushu University Hospital and other affiliated institutes, located in southern Japan. This research clinic was established for treatment of patients with mood disorders and *hikikomori*, and to clarify their underlying multidimensional etiologies, based on the approval of Kyushu University Institutional Review Board for Clinical Research (25–84). To be eligible for the current study, participants had to be at least 15 years old at the time of study enrollment. Clinical patients were informed of the aims and methods regarding the current study and that their participation was completely voluntary. Patients who agreed to study participation completed written informed consent and received a gift card incentive worth approximately US$25. Data were collected between May 2014 and July 2019. During the period, 416 clinical patients participated. We extracted 324 participants that matched the purpose of the present study as follows.

### Cases (*hikikomori*)

Cases consisted of patients with current *hikikomori* conditions. We conducted an original semi‐structured interview for *hikikomori* to evaluate period and severity of social withdrawal. *Hikikomori* was defined as spending most of one's time at home (physical isolation), with duration of at least 6 months. This definition is based on the theoretically and empirically derived criteria we have developed for *hikikomori*.^1^, ^2^

### Clinical controls

Clinical controls consisted of clinical patients without current *hikikomori* conditions. To be included as a clinical control, patients were leaving their homes daily (more than 4 days/week) and they were not restricted to staying indoors. We did not exclude patients who may have had previous *hikikomori* episodes from the clinical controls because this was difficult to evaluate retrospectively.

### Psychological scales

In this pilot study, to assess ASC, we used the Japanese version of the Autism‐Spectrum Quotient (AQ‐J).^39^ The AQ‐J consists of 50 items that are divided into five subscales with 10 questions each. The scale assesses five different areas of cognitive strengths and difficulties related to ASC: Communication, Social Skills, Imagination, Attention to Detail, and Attention Switching. Higher scores on each subscale respectively suggest poor communication skills, poor social skills, poor imagination, exceptional attention to detail, and poor attention switching/strong focus of attention.^40^ Previous studies have proposed cut‐off points of 26, 32, or 33 for the original AQ or the AQ‐J for screening normally intelligent adolescents and adults with PDD.^39^, ^40^, ^41^ We herein set a score of 33 or greater indicating high possibility of ASD based on Wakabayashi *et al*.^39^

The 17‐item Hamilton Depression Rating Scale (HAMD‐17) and the Beck Depression Inventory II (BDI‐II) were also conducted to assess severity of depressive symptoms.^42^, ^43^, ^44^ To further assess other psychological aspects, such as premorbid personality and social functioning, we used the TACS‐22 (score range 0–88), the Japanese version of the abbreviated Lubben Social Network Scale (score range 0–30), the Preference for Solitude Scale (score range 0–20), the Revised UCLA Loneliness Scale (score range 20–80), and the Multidimensional Scale of Perceived Social Support (score range 0–84).^35^, ^45^, ^46^, ^47^, ^48^

### Statistical analyses

We used the Student's *t*‐test and the Mann–Whitney *U*‐test to compare group differences and to assess relations among ASC, psychological traits, personality traits, and psychiatric symptoms.

All analyses were conducted using spss 24 Advanced Statistics (IBM, Armonk, NY, USA) for Mac OS with two‐sided alpha = 0.05.

## Results

Among 416 participants, 103 cases were clinical patients with *hikikomori* conditions and 221 clinical controls were patients without *hikikomori* conditions. Demographic and characteristic data of cases and clinical controls are shown in Table 1. As for marital status, 69.2% (*n* = 72) of cases were single, as were 43.0% (*n* = 95) of clinical controls.

**Table 1:** Characteristics of clinical patients with and without *hikikomori* conditions (*n* = 324)

	Clinical patients with *hikikomori*			Clinical patients without *hikikomori*			Tests of group difference	
	*n*	Mean SD	Median IQR	*n*	Mean SD	Median IQR	Student's *t*‐test, *U*‐test, or χ^2^	*P*‐value
Demographics								
Total (female/male)	103 (42/61)	**—**	**—**	221 (108/113)	**—**	**—**	χ^2^ = 1.850	0.19
Age (years)	**—**	33.02 9.44	**—**	**—**	35.07 9.24	**—**	*t* = −1.845	0.066
Relationship status								
Single	72 (69.2%)	**—**	**—**	95 (43%)	**—**	**—**	**—**	**—**
Committed but not living together	2 (1.9%)	**—**	**—**	11 (5%)	**—**	**—**	**—**	**—**
Living together or married	21 (21.4%)	**—**	**—**	82 (37.1%)	**—**	**—**	**—**	**—**
Divorced	5 (4.9%)	**—**	**—**	22 (10%)	**—**	**—**	**—**	**—**
Education level								
Less than high school	9 (8.7%)	**—**	**—**	12 (5.4%)	**—**	**—**	**—**	**—**
High school degree	35 (34%)	**—**	**—**	62 (28.1%)	**—**	**—**	**—**	**—**
Some college	7 (3.8%)	**—**	**—**	34 (10.9%)	**—**	**—**	**—**	**—**
Bachelor's degree or higher	29 (28.2%)	**—**	**—**	77 (34.9%)	**—**	**—**	**—**	**—**
Clinical symptoms								
Autism‐Spectrum Quotient	**93**	**—**	**27** **8.5**	**196**	**—**	**24** **10.75**	*U* = 6545.5*	**<0.001**
Social skills	**—**	**—**	**7** **3**	**—**	**—**	**5.5** **5**	*U* = 6340*	**<0.001**
Attention switching	**—**	**—**	6 2	**—**	**—**	6 3	*U* = 7902	0.065
Attention to details	**—**	**—**	4 3.5	**—**	**—**	4 4	*U* = 9113.5	0.999
Communication	**—**	**—**	**5** **2**	**—**	**—**	**4** **4**	*U* = 7129*	**0.003**
Imagination	**—**	**—**	**5** **3**	**—**	**—**	**4** **2**	*U* = 7814*	**0.047**
Hamilton Rating Scale for Depression	103	**—**	10 9	219	**—**	10 11	*U* = 10960	0.682
Beck Depression Inventory II	**99**	**25.54** **13.09**	**—**	**212**	**22.23** **12.1**	**—**	*t* = 2.189*	**0.029**
Psychological aspects and social functioning								
Tarumi's Modern‐Type Depression Scale	**101**	**—**	**51** **13**	**215**	**—**	**45** **13**	*U* = 8151*	**<0.001**
Avoidance of Social Roles	**—**	**23.09** **6.06**	**—**	**—**	**20.96** **6.66**	**—**	*t* = 2.73*	**0.007**
Low Self‐Esteem	**—**	**—**	**17** **6**	**—**	**—**	**15** **6**	*U* = 9025*	**0.013**
Complaint	**—**	**—**	10 8	**—**	**—**	9 6	*U* = 9637.5	0.083
Revised UCLA Loneliness Scale	**100**	**43.44** **11.33**	**—**	**207**	**36.56** **10.72**	**—**	*t* = 5.176*	**<0.001**
Multidimensional Scale of Perceived Social Support	**101**	**43.33** **17.09**	**—**	**209**	**54.58** **15.41**	**—**	*t* = −5.813*	**<0.001**
Preference for Solitude	**101**	**—**	**8** **4**	**208**	**—**	**7** **5**	*U* = 8678.5*	**0.013**
Lubben Social Network Scale – 6	101	**—**	**7** **6.5**	**209**	**—**	**10** **8**	*U* = 6224.5*	**<0.001**

Statistically significant results are shown in bold. *P < 0.05. IQR, interquartile range.

**Fig. 1:**
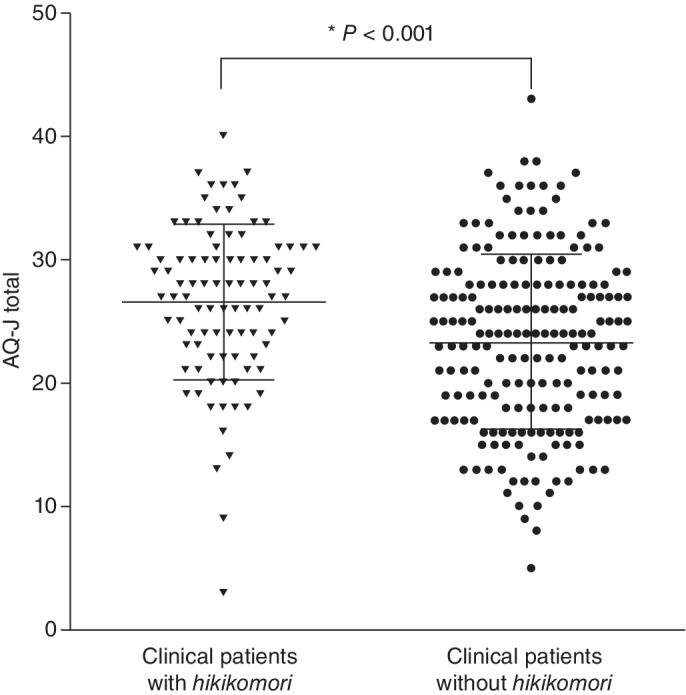
Dot plots of Autism‐Spectrum Quotient, Japanese version (AQ‐J) total scores in cases with *hikikomori* conditions and controls without *hikikomori* conditions. Horizontal bars represent the median values and the interquartile ranges of each group. Statistical *P*‐value was derived from Mann–Whitney *U*‐test. A *P*‐value < 0.05 was considered statistically significant.

For comparisons on *hikikomori* cases and controls, we conducted the Student's *t*‐test and the Mann–Whitney *U*‐test. As for the AQ‐J, a screening questionnaire for ASD, *hikikomori* cases showed higher total scores (*P* < 0.001; Fig. 1). In addition, the cases differed on subscales in terms of cognitive and social functioning, including having fewer social skills (*P* < 0.001), less communication (*P* = 0.003), and less imagination (*P* = 0.047). Regarding the severity of depressive symptoms, *hikikomori* cases showed significantly higher scores on a self‐rating scale of the BDI‐II (*P* = 0.029), while an interview‐based score for the HAMD‐17 showed no significant difference.

*Hikikomori* cases showed higher total scores on the TACS‐22, a scale for evaluating premorbid traits of MTD (*P* < 0.001).^35^ Cases were also significant in having more traits of MTD, including avoidance of social roles (*P* = 0.007) and low self‐esteem (*P* = 0.013). As for social support and social network, *hikikomori* cases differed in terms of solitude and loneliness in having more preference for solitude (*P* = 0.013) while feeling lonelier (*P* < 0.001).

**Fig. 2:**
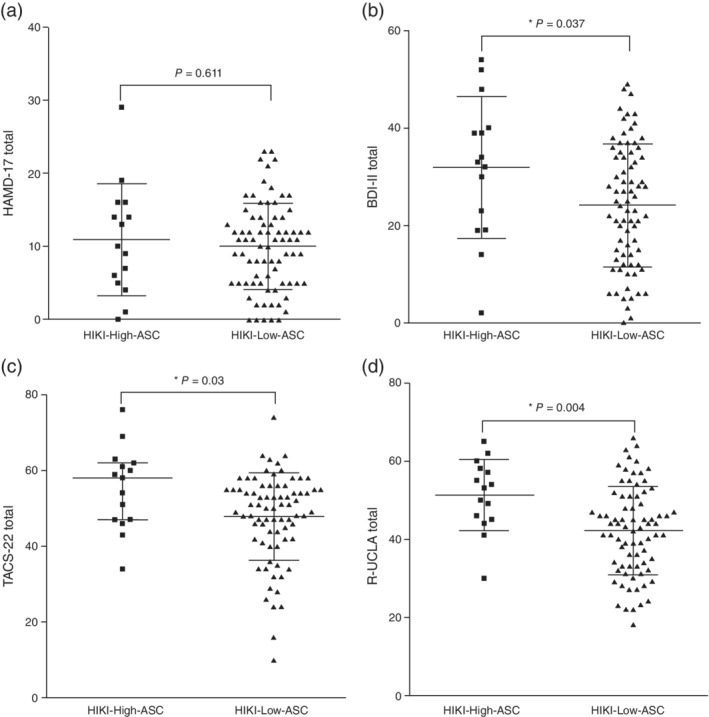
Comparison of psychological scales in *hikikomori* cases with higher autism spectrum conditions (HIKI‐High‐ASC) and *hikikomori* cases with lower autism spectrum conditions (HIKI‐Low‐ASC). A *P*‐value < 0.05 was considered statistically significant. (a) Dot plots of 17‐item Hamilton Depression Rating Scale (HAMD‐17) total scores in each group with horizontal bars representing the median values and the interquartile ranges. Statistical *P*‐value was derived from Mann–Whitney *U*‐test. (b) Dot plots of Beck Depression Inventory II (BDI‐II) total scores in each group with horizontal bars representing the mean values and the SD. Statistical *P*‐value was derived from Student's *t*‐test. (c) Dot plots of 22‐item Tarumi's Modern‐Type Depression Trait Scale: Avoidance of Social Roles, Complaint, and Low Self‐Esteem (TACS‐22) total scores in each group with horizontal bars representing the median values and the interquartile ranges. Statistical *P*‐value was derived from Mann–Whitney *U*‐test. (d) Dot plots of Revised UCLA Loneliness Scale (R‐UCLA) total scores in each group with horizontal bars representing the mean values and the SD. Statistical *P*‐value was derived from Student's *t*‐test.

Next, we compared among the *hikikomori* patients based on the cut‐off score of 33 on the AQ‐J, in order to clarify the differences between 15 *hikikomori* cases with higher ASC (HIKI‐High‐ASC) and 78 *hikikomori* cases with lower ASC (HIKI‐Low‐ASC; Table 2). HIKI‐High‐ASC showed higher total AQ‐J scores. The group also showed higher scores for Attention Switching (*P* < 0.001), Social Skills (*P* < 0.001), Communication (*P* < 0.001), and Imagination (*P* = 0.004). HIKI‐High‐ASC showed higher BDI‐II scores compared to HIKI‐Low‐ASC (*P* = 0.037), while showing no significant difference on the HAMD‐17 (Fig. 2a,b). Regarding MTD traits, HIKI‐High‐ASC showed higher scores for Low Self‐Esteem (*P* = 0.005) and Complaint Tendencies (*P* = 0.044) on the TACS‐22 (Fig. 2c). As for social relationships and networks, HIKI‐High‐ASC were more likely to prefer solitude (*P* = 0.022) while feeling lonelier (*P* = 0.004; Fig. 2d), and had smaller social networks (*P* = 0.017) and less social support (*P* = 0.036).

**Table 2:** Characteristics of clinical *hikikomori* patients with higher or lower autism spectrum conditions (*n* = 93)

	*Hikikomori* patients with higher autism spectrum conditions			*Hikikomori* patients with lower autism spectrum conditions			Tests of group difference	
	*n*	Mean SD	Median IQR	*n*	Mean SD	Median IQR	Student's *t*‐test, *U*‐test, or χ^2^	*P*‐value
Demographics								
Total (female/male)	15 (4/11)	**—**	**—**	78 (34/44)	**—**	**—**	χ^2^ = 1.491	0.26
Age (years)	**—**	31 8.9	**—**	**—**	33.09 9.44	**—**	*t* = −0.792	0.43
Clinical symptoms								
Autism‐Spectrum Quotient	**15**	**—**	**35** **3**	**78**	**—**	**26** **7.5**	*U* = 0*	**<0.001**
Social Skills	**—**	**—**	**9** **2**	**—**	**—**	**7** **2.25**	*U* = 167*	**<0.001**
Attention Switching	**—**	**—**	**8** **2**	**—**	**—**	**5.5** **2.25**	*U* = 206.5*	**<0.001**
Attention to Details	**—**	**—**	5 4	**—**	**—**	4 3	*U* = 449	0.152
Communication	**—**	**—**	**8** **3**	**—**	**—**	**5** **2**	*U* = 157*	**<0.001**
Imagination	**—**	**6** **1.65**	**—**	**—**	**4.37** **2.0**	**—**	*t* = 2.068*	**0.004**
Hamilton Rating Scale for Depression	15	10.87 7.62	**—**	78	9.97 5.90	**—**	*t* = 0.511	0.611
Beck Depression Inventory II	**15**	**31.87** **14.53**	**—**	**78**	**24.12** **12.65**	**—**	*t* = 2.116*	**0.037**
Psychological aspects and social functioning								
Tarumi's Modern‐Type Depression Scale	**15**	**—**	**58** **15**	**77**	**—**	**50** **13**	*U* = 373*	**0.03**
Avoidance of Social Roles	**—**	23.67 6.68	**—**	**—**	22.76 5.95	**—**	*t* = 0.533	0.595
Low Self‐Esteem	**—**	**—**	**20** **6**	**—**	**—**	**16** **5**	*U* = 313*	**0.005**
Complaint	**—**	**—**	**12** **8**	**—**	**—**	**10** **8**	*U* = 387*	**0.044**
Revised UCLA Loneliness Scale	**15**	**51.27** **9.13**	**—**	**77**	**42.21** **11.30**	**—**	*t* = 2.92*	**0.004**
Multidimensional Scale of Perceived Social Support	**15**	**—**	**35** **16**	**78**	**—**	**46.5** **27**	*U* = 384.5*	**0.036**
Preference for Solitude	**15**	**—**	**9** **3**	**78**	**—**	**8** **4.25**	*U* = 366.5*	**0.022**
Lubben Social Network Scale – 6	**15**	**—**	**4** **6**	**78**	**—**	**7** **6**	*U* = 357.5*	**0.017**

Statistically significant results are shown in bold. *P < 0.05. IQR, interquartile range.

## Discussion

In the present study, we have revealed some interesting relations between *hikikomori* and ASD, including undiagnosed ASC.

When compared to clinical controls without *hikikomori* conditions, *hikikomori* cases had significantly higher scores on AQ‐J showing fewer social skills, less communication, and less imagination, and this data suggests that *hikikomori* sufferers are more likely to have autistic tendencies. Moreover, *hikikomori* sufferers with ASC can be mostly understood as having difficulty in social communication and social interaction, related to Criteria A in the DSM‐5.^24^ Accordingly, we suppose an individual may come to withdraw himself/herself because he/she feels burdened to interact with others.

Here we showed that *hikikomori* cases, especially those with high ASC, have weakness in attention switching in addition to social skills, communication, and imagination. ‘Attention switching’ can be described as the cognitive ability for multitasking and changing one's approach as needed, and difficulty in the ability may be associated with excessive persistence. Thus, those aspects should be considered when providing support for social participation and employment for such individuals.

Regarding depressive symptoms, *hikikomori* cases showed significantly higher total scores on the self‐rated BDI‐II compared to controls without *hikikomori* conditions, while the two groups did not show difference on the semi‐structured interview‐based HAMD‐17. Moreover, HIKI‐High‐ASC were significantly higher than HIKI‐Low‐ASC on the self‐rated BDI‐II scores. These findings suggest *hikikomori* sufferers are likely to evaluate their own depressive symptoms more severely, and this tendency is especially high when they have higher ASC. *Hikikomori* sufferers with high ASC may have difficulty in identifying their depressive feelings so that they tend to underestimate/overestimate their own symptoms.

Interestingly, another notable condition that shows a distinguishable gap between subjective and objective evaluations of one's own depressive symptoms is MTD. We have recently proposed that MTD could be a gateway disorder to *hikikomori*.^28^ Supporting our hypothesis, the present study has revealed that MTD traits were significantly higher in *hikikomori* patients compared to patients without *hikikomori* conditions.

Moreover, we have revealed that *hikikomori* sufferers with high ASC had significantly lower self‐esteem and higher complaint tendencies, and such aspects could be regarded as vulnerable narcissism, which is also a typical trait of MTD.^29^ Lai and Baron‐Cohen have discussed differences between ASD and narcissistic personality disorder in terms of their similar egocentricity; narcissistic personality disorder is characterized by reduced affective empathy and sympathy that leads to indifference toward others, whereas individuals with ASC are suggested to be often unaware of the social effect of their behavior because of a deficit in cognitive empathy.^49^

Reduced self‐esteem in *hikikomori* sufferers with high ASC may be associated with: (i) self‐awareness of impaired social skills; and (ii) repeated negative social interactions. Kuusikko *et al*. have suggested that children and adolescents with high‐functioning autism are likely to become self‐conscious about their competency in social situations as they begin to recognize their own impaired social skills.^50^ In addition, previous research has suggested a higher frequency of experiencing bullying or peer rejection, and these negative social interactions may be related to greater risk of *hikikomori*.^32^, ^33^, ^34^

Based on the present study, we suggest that *hikikomori* sufferers with high ASC may have difficulties in introspectively knowing themselves and solving problems because of dysfunctions in the above‐mentioned social interactions, which often lead to extrapunitive coping styles, where others surrounding the individuals are easily blamed for the problems that are caused by the individuals themselves. Thus, *hikikomori* sufferers with ASC should be supported with a focus on enhancing self‐esteem, empowering skills to seek support, and helping such individuals reduce anxiety, stress, and vulnerability to change.

There are some limitations in the present study. First, this study used a cross‐sectional research design, thus we did not come to a conclusion on the causal relation underlying *hikikomori* and ASC. Therefore, longitudinal research is needed to confirm the present findings.

Second, we used the AQ‐J alone to assess ASC in the present study. However, Kurita and Koyama suggest that the AQ‐J measures general mental health problems other than autistic traits.^41^ Therefore, assessment relying on the AQ‐J alone may limit the accuracy of picking up aspects of ASC. Thus, future work with comprehensive evaluations, including IQ, responsiveness in social situations, social maturity, sensory processing patterns, and diagnosis of ASD, such as the Autism Diagnostic Observation Schedule, Second Edition, should be conducted.^51^, ^52^

### Conclusion

In this study, we have explored the relations between *hikikomori* and ASD (including undiagnosed ASC), and their associations with depressive symptoms, especially MTD. Interestingly, our results suggest that *hikikomori* sufferers are more likely to have autistic tendencies and that *hikikomori* sufferers with high ASC may have much more difficulty in social communication and social interaction. Those with high ASC may also have lower self‐esteem and higher complaint tendencies as aspects of MTD traits. Reduced self‐esteem may be related to self‐awareness of impaired social skills and experiencing negative social interactions as a result of difficulty in social communication. MTD traits in persons with ASC could attribute to greater risk of *hikikomori*. Further investigation to validate our findings with comprehensive evaluation systems of ASD is strongly warranted.

## Disclosure statement

All authors report no financial relationships with commercial interests.

## Author contributions

T.A.K. contributed to the conception and design. T.A.K. and R.K. were responsible for protocol of the investigation. K.K., K.H., N.K., H.K., R.K., and T.A.K. contributed to the clinical data collection. R.K., M.T., H.K., S.K., and T.A.K. contributed to the data checking, analysis, and interpretation of data. R.K. drafted the manuscript, and M.T., H.K., S.K., and T.A.K. revised it critically for important intellectual content. All authors have provided final approval of the version to be published.
